# Evaluation and dynamic evolution of maternal and child health services in township health centers in ethnic minority areas of Guangxi, China

**DOI:** 10.1017/S1463423625100224

**Published:** 2025-07-22

**Authors:** Zhuanzhi Tang, Ranfeng Hang, Siyuan Wang, Jianying Liu, Wuxiang Shi

**Affiliations:** 1 School of Public Administration, Northwest University, Xi’an, Shaanxi 710127, PR China; 2 School of Public Health, Guilin Medical University, Guilin, Guangxi 541001, PR China; 3 Labour Union, Guilin Medical University, Guilin, Guangxi 541001, PR China; 4 School of Humanities and Management, Guilin Medical University, Guilin, Guangxi 541001, PR China

**Keywords:** DEA, entropy weight coefficient method, Malmquist index, maternal and child services, township health centers

## Abstract

**Aim:**

To achieve more efficient and comprehensive maternal and child health (MCH) care services in rural areas through optimizing resource allocation and enhancing service quality.

**Background:**

With the increasing awareness of health among rural residents and the growing demand for MCH care, township health centers, as a crucial component of primary medical services, have emerged as a key factor in ensuring the health of women and children in rural areas.

**Methods:**

Using a multi-stage stratified random sampling method, this study conducted on-site investigations on 49 township health centers across six districts and counties of Guilin, Guangxi. Descriptive statistics, entropy weight coefficient method, Data Envelopment Analysis (DEA) Banker–Charnes–Cooper (BCC) Model and Malmquist index were employed for dynamic analysis.

**Findings:**

The results indicate an upward trend in the incidence rates of birth defects and low birth weight in MCH services. Disparities in efficiency across regions are observed, which are associated with the economic status and capacity of MCH services in each area. Dynamic results from the Malmquist index show that the total factor productivity of MCH services experienced an upward trend from 2016 to 2021, with efficiency primarily influenced by scale efficiency. Updating management concepts is crucial for effectively addressing the relationship between scaling up and quality improvement.

## Background

The health status of women and children directly reflect the level of national health and social development (Lebrun-Harris *et al.*, 2022). In the eight Millennium Development Goals (MDGs) advocated by the United Nations, two MDGs are directly related to maternal and child health (MCH): MDG 4 (a two-third reduction in child mortality between 1990 and 2015) and MDG 5 (a three-quarter reduction in maternal mortality ratio between 1990 and 2015) (Zhang *et al.*, 2020). Being a United Nations member, the Chinese government formulated a series of policies and goals, such as the “Healthy China 2030” plan (2016) (Ning *et al.*, 2024), the China National Program for Women’s Development (2021–2030) and the China National Program for Child Development (2021–2030) (Chen *et al.*, 2019), which place higher demands for MCH than set by the United Nations. Many studies found the temporal trends of MCH indicators in China have a remarkable improvement over the past few decades (Qiao *et al.*, 2021; Yip *et al.*, 2023). For example, MDG 4, which aims to reduce child mortality by two-thirds, has been reached in advance of 9 years; MDG 5, which seeks to decrease maternal mortality by three-quarters, has been achieved 1 year ahead of schedule (Campbell, 2017; Li *et al.*, 2017).

However, unequal and inefficient MCH remains a persistent issue in China, particularly in rural areas (Gebremeskel *et al.*, 2023; Yu *et al.*, 2022). In rural China, the number of “left-behind women” has reached 47 million, with 59.04% of the 75.53 million children under the age of 5 residing in rural areas (Sun *et al.*, 2020; Xue *et al.*, 2021). There are large differences in the use of MCH between richer and poorer regions, between urban and rural areas. In 2018, the under-five mortality rate in urban areas was 4.4‰, while in rural areas it reached 10.2‰. Additionally, the maternal mortality rate in urban areas was 15.5 per 100,000, whereas in rural areas it was 19.9 per 100,000 (Dai and Menhas, 2020; Yu *et al.*, 2023). The efficiency of MCH services in rural areas is also much lower than in urban areas (Rizqi and Kurniawan, 2023). The Chinese government has implemented a series of measures aimed at enhancing the quality of MCH services for vulnerable populations in underdeveloped rural areas (Zhao *et al.*, 2019). Including “Reduce Maternal Mortality Ratio and Eliminate Neonatal Tetanus,” (or Jiang Xiao Project), “The New Rural Cooperative Medical System,” “Subsidize Hospital Childbirths for Rural Women,” and so on. These programs and policies have directly and indirectly improved MCH in the central and western rural areas (Zhang *et al.*, 2015). However, MCH outcomes in rural minority areas in western China remain poor (Gao *et al.*, 2017).

Guangxi (20 54 ′-26 20′ N, 104 26 ′-112 04′ E), located in western China, is one of the five major ethnic minority autonomous regions in China. According to the seventh census of China, the population of Guangxi is 50.1268 million, making it the most populous among ethnic minority province in the country, which is often considered an economically underdeveloped region based on China’s provincial Gross Domestic Product (GDP) per capita ranking (Xu *et al.*, 2022). Research indicates that in 2017, the quality evaluation of MCH in Guangxi ranked 19th among 31 provinces for maternal health care, while children’s health care was ranked 31st (Li *et al.*, 2023). The Data Envelopment Analysis (DEA) efficiency evaluation value for MCH institutions at the municipal level in Guangxi was 0.953 (Yao *et al.*, 2023), significantly lower than that of developed regions such as Shanghai at 1.000 (Cai *et al.*, 2022), Beijing at 0.981 (Zhao and Zheng, 2023) and Guangdong at 1.031 (Li *et al.*, 2019). This shows that there is considerable room for improvement in MCH for ethnic minorities in Guangxi. As the gatekeepers of MCH in rural areas, township health centers are the primary choice for mothers and children seeking health care services. In 2021, China began implementing the “Comprehensive Three-Child Policy” (Zhu *et al.*, 2024). With the increased number of elderly “second-child” pregnant women, the number of high-risk and premature babies has also increased, placing immense pressure on MCH services in township health centers (Tian *et al.*, 2023; Zhang *et al.*, 2024). Therefore, the study of MCH in township health centers plays a key role in improving MCH care in rural areas.

However, previous studies have primarily focused on MCH at the provincial and municipal levels (Huang *et al.*, 2020; Cai *et al.*, 2022) and at the district and county levels (Zhang *et al.*, 2020), using cross-sectional research designs. In terms of efficiency measurement, the main methods employed include the weighted RSR method and fuzzy comprehensive evaluation (Zhao *et al.*, 2021), as well as Banker–Charnes–Cooper (BCC)-DEA and Charnes–Cooper–Rhodes (CCR)-DEA efficiency evaluation models (Değirmenci, 2021; Ekinci, 2024), and the Technique for Order Preference by Similarity to Ideal Solution (TOPSIS) method (Selamzade and Ersoy, 2023). Additionally, there has been limited observation of changes in MCH over time (Trakakis *et al.*, 2021). Furthermore, research on MCH in township health centers in impoverished ethnic minority areas is very limited (Yan *et al.*, 2020). Therefore, it is essential to conduct a comprehensive assessment of MCH in rural township health centers across different regions of China over a period of time.

Based on the above reasons, this study collected 6 years of longitudinal data, and used entropy weight coefficient method, BCC-DEA Model and Malmquist index Model to comprehensively evaluate and analyze the current situation, trend and efficiency of MCH in township health centers in minority areas. Our research aims to achieve the following objectives: First, to evaluate the current status and trends of MCH services. Second, to assess the efficiency of MCH services in township health centers using the BCC-DEA model, and to conduct a dynamic analysis of total factor productivity based on the Malmquist index model. Third, to provide targeted recommendations for improving the quality of MCH services in rural township health centers based on the research findings. The research findings offer valuable perspectives for policymakers and healthcare stakeholders in Guangxi, China, underscoring disparities in efficiency levels and illuminating potential avenues for enhancement. Through remedying these inefficiencies, policymakers can augment the efficiency of MCH in township health centers, ultimately improving the health of mothers and children in ethnic minority rural areas.

## Materials and methods

### Data sources and indicators selection

A multi-stage stratified random sampling method was employed to select a total of 49 township health centers for on-site investigation, including Lingui District, Lingchuan County, Yangshuo County, Gongcheng Yao Autonomous County, Longsheng Various Nationalities Autonomous County, and Yanshan District. Primary data sources for this study included the MCH Annual Reports and the “Gui Fu Er” information system from each township health center. Based on the “China Women and Children Development Plan 2011-2020” and in conjunction with a review of relevant literature (Duff *et al.*, 2021; Esquivel *et al.*, 2014), considering the importance and availability of indicators, relevant indicators that could reflect the capacity of MCH services were identified for final use.

In the assessment of the current situation, we reference previous study results (Qin and Zhu, 2023; Wu *et al.*, 2023; Zhao *et al.*, 2021). The indicators included rates of early pregnancy health check-ups, prenatal examinations, postnatal visits, systematic management of pregnant women, high-risk maternal management, hospitalization for childbirth, maternal mortality, moderate to severe anemia among pregnant women, prenatal screening, exclusive breastfeeding for infants aged 0-6 months, neonatal mortality, mortality of children under 5, newborn visits, systematic management of children under 3, systematic management of children under 7, premarital medical check-ups, birth defects, and low birth weight (corresponding to X1-X18). In terms of input and output, based on previous research (Cheng *et al.*, 2024; Huang *et al.*, 2020; Li *et al.*, 2019), the number of full-time MCH care workers and equipment worth over 10,000 yuan are used as indicators for human resources and capital. The rates of systematic management for pregnant women and for children under 3 years old represent the outcomes of MCH services.

### Data analysis

#### Entropy weight coefficient method

The entropy weight coefficient method is an objective weighting approach that determines the weight of the total score based on the data variance of individual unit scores, effectively avoiding subjective errors from evaluators. In this study, an improvement was made to the entropy weight coefficient method, incorporating time indicators for comparison between different years, based on relevant literature (Kohl *et al.*, 2019; Zou *et al.*, 2023). The entropy weight method calculation includes the following six steps, as detailed in the Appendix.

#### BCC-DEA model

This study adopts the DEA method to measure MCH efficiency. DEA is an analytical method proposed by scholars such as Charnes and Cooper (1978) for assessing the relative efficiency among Decision Making Units with multiple inputs and outputs (Ratner *et al.*, 2023). Since the input-output indicators of relevant healthcare resources exhibit variable returns to scale, the study uses the input-oriented BCC-DEA model, and the specific calculation formulas are provided in the Appendix.

#### Malmquist index model

Traditional DEA model primarily facilitate static assessments of resource allocation efficiency using cross-sectional data (Nazarko, 2024). However, this study spans a longer time frame, necessitating consideration of temporal variations. The Malmquist index provides a means to evaluate changes in efficiency over a specified period, reflecting the performance of decision-making units (Andrejić *et al.*, 2021). Consequently, we implemented the Malmquist Productivity Index method to analyze panel data and illustrate dynamic shifts in efficiency. The MPI includes input-oriented efficiency change (EFFCH) and technical change (TECHCH). Efficiency change can also be divided into scale efficiency change (SECH) and pure efficiency change (PECH). The MPI, also known as Total Factor Productivity Changes (TFPCH), is derived from the distance function and can be represented by the following mathematical equations (The specific calculation formulas are provided in the Appendix).

## Results

### Maternal and child health service implementation

Table 1 shows that from 2016 to 2021, MCH indicators, such as the high-risk maternal management rate, hospitalization rate for childbirth, prenatal screening rate, and premarital check-up rate, exhibited an upward trend. Meanwhile, the neonatal mortality rate, under-five mortality rate, and the systematic management rate for children under 7 years showed a downward trend. The breastfeeding rate for infants aged 0–6 months demonstrated a fluctuating downward trend, while the incidence rate of birth defects and the incidence rate of low birth weight displayed an upward trend.

**Table 1.** Maternal and child health services, 2016–2021

Years Items	2016	2017	2018	2019	2020	2021
X1_#_ (%)	98.6	96.5	97.8	97.9	98.2	97.1
X2_#_ (%)	99.5	99.3	98.9	97.8	98.0	98.4
X3_#_ (%)	98.4	98.5	97.9	97.6	97.7	97.3
X4_#_ (%)	97.5	96.5	96.4	96.7	96.2	97.2
X5_#_ (%)	99.0	99.4	99.7	99.9	100.0	100.0
X6_#_ (%)	100.0	100.0	100.0	100.0	100.0	100.0
X7_*_ (‰)	0.0	0.0	0.0	0.7	0.0	0.9
X8_*_ (%)	1.0	1.1	1.1	1.1	1.0	1.0
X9_#_ (%)	83.8	84.6	86.6	87.2	87.5	87.7
X10_+_ (%)	88.7	91.2	91.8	90.7	88.5	87.7
X11- (‰)	4.1	4.2	3.8	2.9	2.5	2.1
X12- (‰)	99.2	99.4	99.4	99.6	99.7	99.2
X13_+_ (%)	99.2	99.4	99.4	99.6	99.7	99.2
X14_+_ (%)	93.2	92.6	93.2	94.2	95.0	95.5
X15_+_ (%)	94.9	92.4	93.7	94.8	92.3	93.3
X16_#_ (%)	98.9	98.8	99.8	99.5	99.6	99.7
X17- (‰)	11.2	10.5	12.0	12.6	14.0	14.2
X18- (%)	4.3	5.4	5.6	5.8	5.2	5.1

*Note*: Maternal Health Care: _#_ represents positive indicators, _*_ represents negative indicators; Child Health Care (under 7 years old): _+_ represents positive indicators, - represents negative indicators.

### Maternal health entropy weight coefficient score

**Figure 1.**
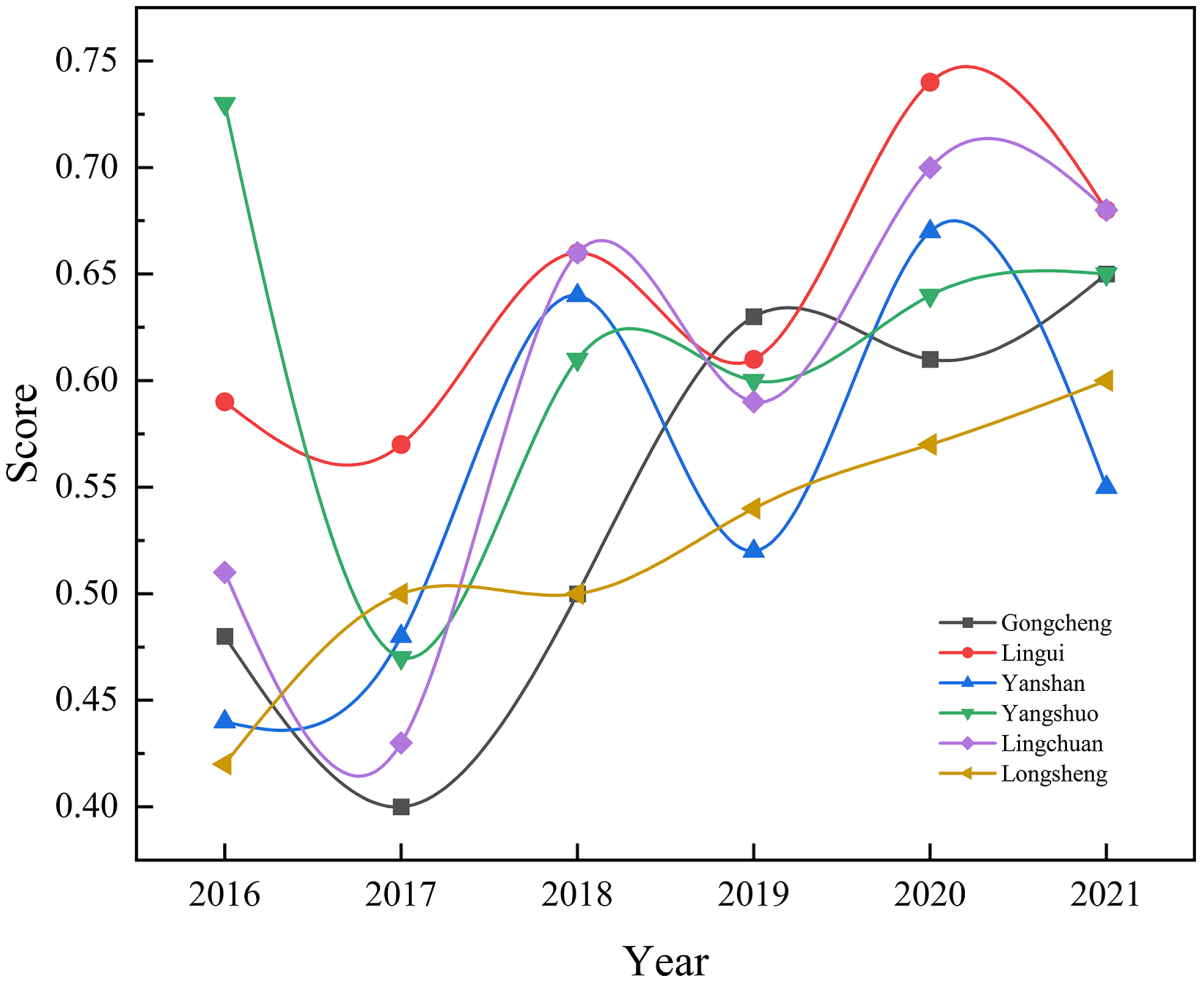
Changes in maternal health scores by region from 2016 to 2021.

Figure 1 shows the maternal health care scores of township hospitals across various regions from 2016 to 2021. Overall, the scores exhibited a dynamic upward trend, although differences among regions were observed. Specifically, in 2017, the scores reached their lowest point across all regions, with Gongcheng Yao Autonomous County scoring the lowest, below 0.4, indicating a gap in maternal care compared to other regions. In 2020, the maternal care scores peaked, with Lingui District scoring the highest, above 0.7, reflecting the best performance in maternal health care. In comparison, Longsheng Autonomous County and Gongcheng Yao Autonomous County scored lower than other regions, suggesting room for improvement in maternal care in these areas.

### Health entropy weight coefficient scores for children under 7 years of age

**Figure 2.**
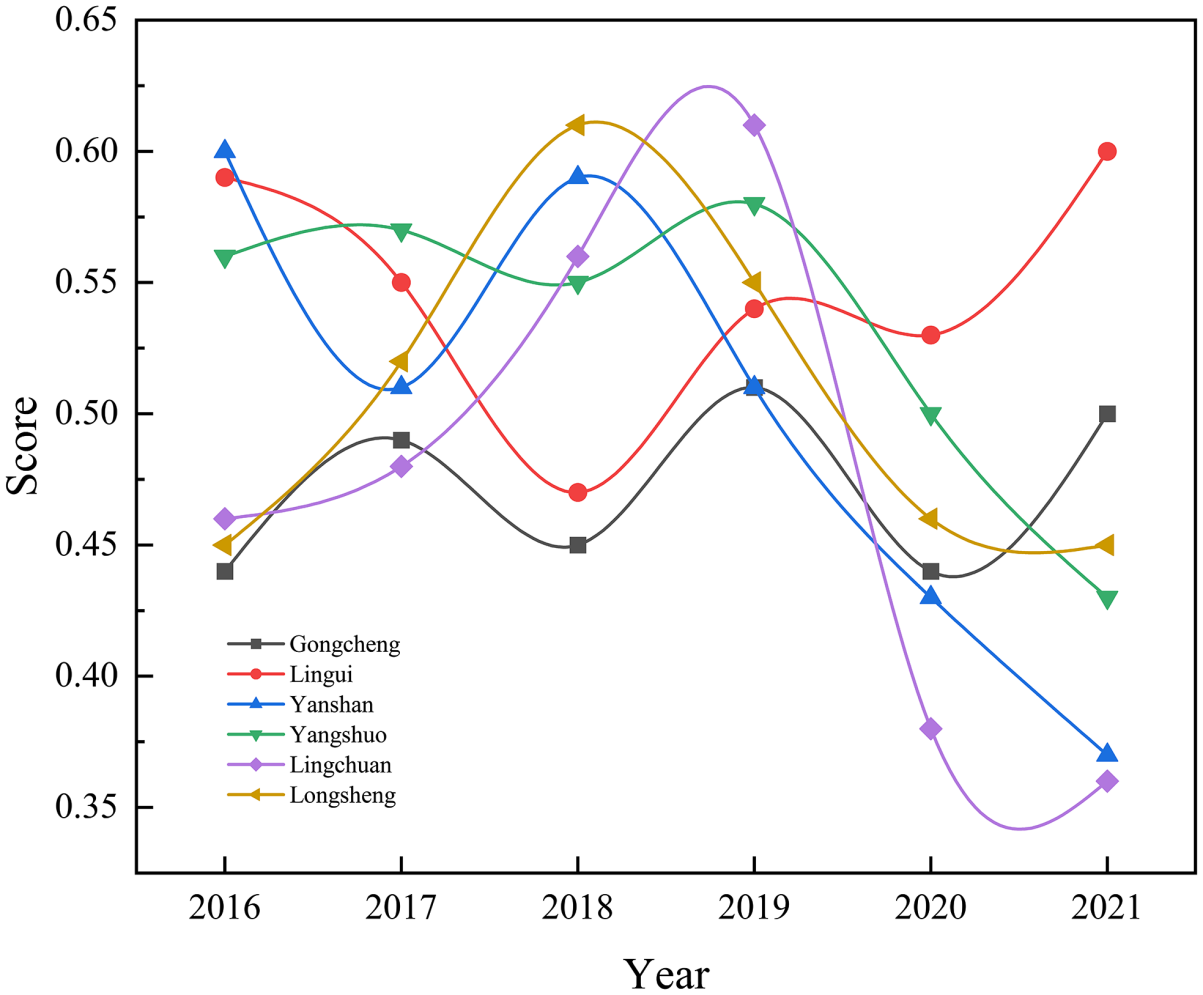
Changes in health scores for children under 7 years of age by region from 2016 to 2021.

Figure 2 illustrates the health care scores for children under seven years old in township hospitals across different regions from 2016 to 2021. Overall, the scores showed a dynamic downward trend, with significant differences among regions. Specifically, in 2020, the scores for children’s health care were at their lowest, with Lingchuan County experiencing a sharp decline compared to 2019. This highlights the urgent need to address children’s health care in that area. However, by 2021, the scores in Lingui District and Gongcheng Yao Autonomous County had improved to some extent, while other regions continued to experience a decline, with Lingchuan County scoring the lowest.

### Efficiency of maternal and child health services from 2016 to 2021

#### Basic information of indicators

As shown in Table 2, the average number of full-time MCH care workers increased from 20.50 in 2016 to 21.33 in 2021, representing a 4.05% growth. The average number of equipment valued at over 10,000 yuan rose from 127.50 units in 2016 to 286.67 units in 2021, a growth of 124.84%. This indicates that China has increased its investment in healthcare resources for primary healthcare institutions. The rate of systematic management of pregnant women showed a fluctuating downward trend, while the systematic management rate for children under three years old exhibited a dynamic upward trend. Overall, the increase in input and output has not been entirely balanced, indicating a need for further optimization and improvement. The large gap between the maximum and minimum values of indicators also reflects widening regional disparities.

**Table 2.** Input-output data from 2016 to 2021

Years	Statistics	Input		Output	
		Number of full-time maternal and children healthcare workers	Number of equipment over 10,000 yuan	Rate of systematic management of pregnant women	Rate of systematic management for children under 3 years old
2016	Max	29.00	215.00	98.39	95.41
	Minimum	8.00	36.00	96.21	91.49
	Mean	20.50	127.50	97.21	93.60
	Median	23.00	146.00	97.20	93.70
2017	Max	29.00	247.00	97.99	93.78
	Minimum	8.00	38.00	94.58	90.94
	Mean	20.33	144.17	96.36	92.64
	Median	23.00	163.50	96.57	92.79
2018	Max	28.00	269.00	98.63	93.82
	Minimum	7.00	39.00	94.21	91.46
	Mean	20.33	154.67	96.44	93.14
	Median	23.50	178.00	96.69	93.43
2019	Max	27.00	269.00	97.74	95.54
	Minimum	8.00	43.00	95.82	91.32
	Mean	20.67	176.00	97.11	94.05
	Median	24.00	197.50	97.42	94.37
2020	Max	27.00	315.00	97.33	96.53
	Minimum	10.00	45.00	95.49	92.29
	Mean	21.00	222.17	96.53	94.87
	Median	24.00	280.50	96.77	95.49
2021	Max	27.00	444.00	98.13	96.95
	Minimum	12.00	65.00	96.54	93.74
	Mean	21.33	286.67	97.20	95.45
	Median	24.50	344.50	97.05	95.81

### Static analysis of service efficiency from 2016 to 2021 based on the BCC-DEA model

As shown in Table 3, healthcare service outputs were effective under DEA in 2016 and 2018, while they were deemed ineffective in 2017 and from 2019 to 2021. All the ineffective years are due to scale efficiency being less than 1. Considering returns to scale, scale returns remained constant in 2016 and 2018. In 2017, scale returns exhibited an increasing trend, indicating that the rate of increase in healthcare resource input was lower than the rate of output growth. This suggests the potential for further scientifically and reasonably increasing the proportion of resource input and appropriately expanding scale to achieve greater output. Scale returns for 2019 to 2021 showed a decreasing trend, signifying that the growth rate of healthcare resource input exceeded the growth rate of output during these three years, indicating the necessity to appropriately reduce the scale of resource allocation.

**Table 3.** Static results of service efficiency from 2016 to 2021

Years	PTE	SE	OE(θ)	S ^−^	S ^+^	Returns to Scale
2016	1.000	1.000	1.000	0.000	0.000	Constant
2017	1.000	0.999	0.999	0.000	0.318	Decreasing
2018	1.000	1.000	1.000	0.000	0.000	Constant
2019	1.000	0.993	0.993	112.052	0.272	Decreasing
2020	1.000	0.986	0.986	369.419	1.701	Decreasing
2021	1.000	0.977	0.977	729.018	1.632	Decreasing

PTE: Pure technical efficiency, SE: Scale efficiency, OE: Overall efficiency.

### Static analysis of service efficiency in various regions in 2021

As shown in Table 4, from the efficiency analysis of different regions, it is evident that only Yanshan District exhibited effective service output under DEA. Gongcheng County, Lingui District, Yangshuo County, and Longsheng County are ineffective due to scale efficiency being less than 1. In contrast, Lingchuan is ineffective because both pure technical efficiency and scale efficiency are less than 1. Considering returns to scale, Yanshan District maintained constant scale returns. Gongcheng County, Lingui District, Yangshuo County, and Longsheng County demonstrated decreasing scale returns, indicating the need to appropriately reduce the scale of resource allocation. Lingchuan County exhibited increasing scale returns, suggesting the potential to expand scale appropriately to achieve greater output.

**Table 4.** Static results of service efficiency in various regions in 2021

areas	PTE	SE	OE(θ)	S ^−^	S ^+^	Returns to Scale
Gongcheng	1.000	0.488	0.488	128.683	0.213	Decreasing
Lingui	1.000	0.458	0.458	136.254	1.795	Decreasing
Yanshan	1.000	1.000	1.000	0.000	0.000	Constant
Yangshuo	1.000	0.876	0.876	253.28	1.425	Decreasing
Lingchuan	0.500	0.998	0.499	96.85	0.274	Increasing
Longsheng	1.000	0.538	0.538	1.771	1.695	Decreasing

PTE: Pure technical efficiency, SE: Scale efficiency, OE: Overall efficiency.

### An overall analysis of malmquist index for maternal and child health services

**Figure 3.**
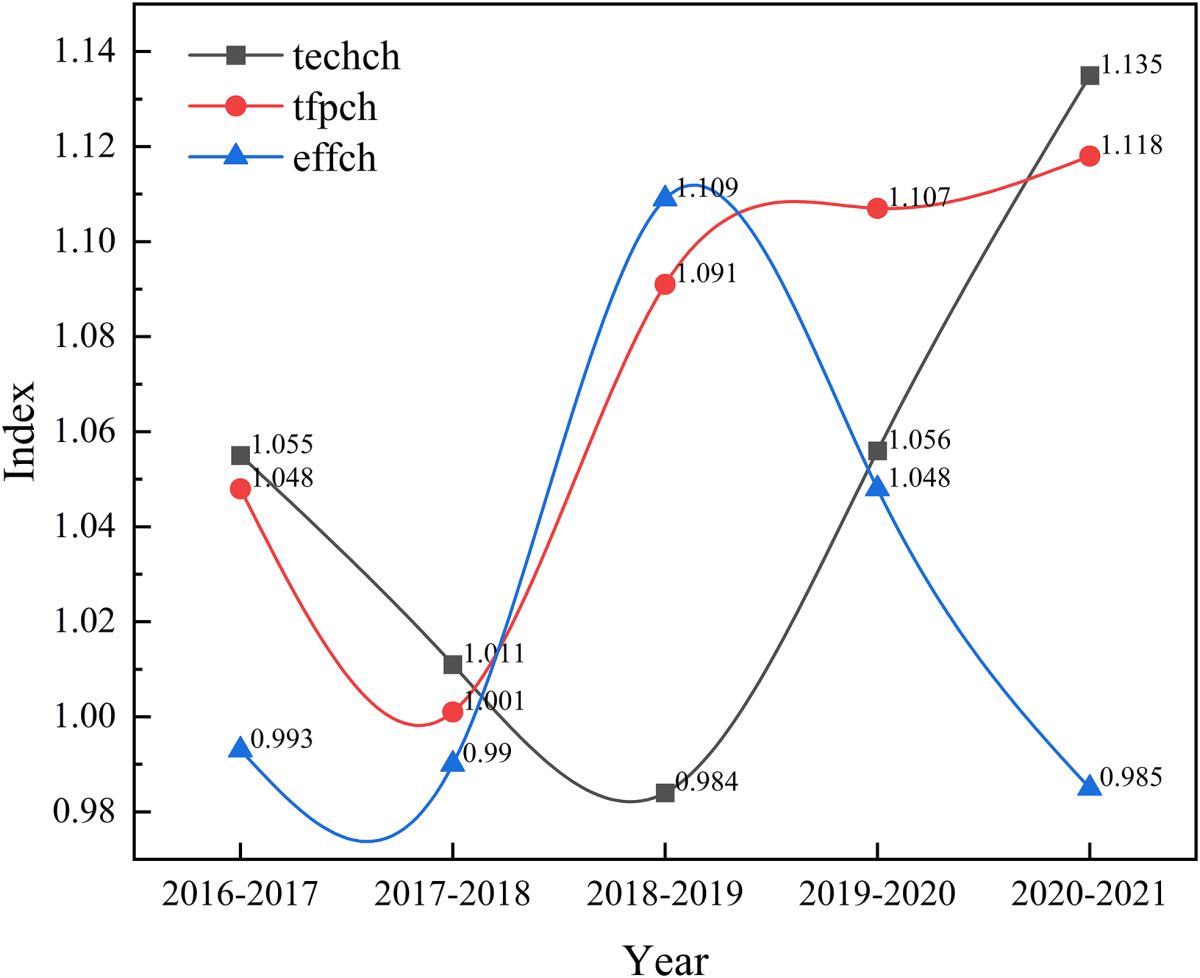
Changes in total factor productivity of maternal and child health services from 2016 to 2021.

As shown in Figure 3, from 2016 to 2021, total factor productivity exhibited a dynamic upward trend, indicating an overall improvement in resource allocation efficiency during this period. The average annual growth rate of total factor productivity from 2016 to 2021 was 7.3%. An analysis of the components of total factor productivity reveals that the efficiency values were greater than 1 from 2016 to 2018 and from 2020 to 2021, primarily due to an increase in the technological change index. In 2018-2019, the efficiency value was greater than 1 primarily due to an increase in the efficiency change. In 2019-2020, the efficiency value was greater than 1, mainly due to both the technological change index and the efficiency change index being greater than 1.

**Figure 4.**
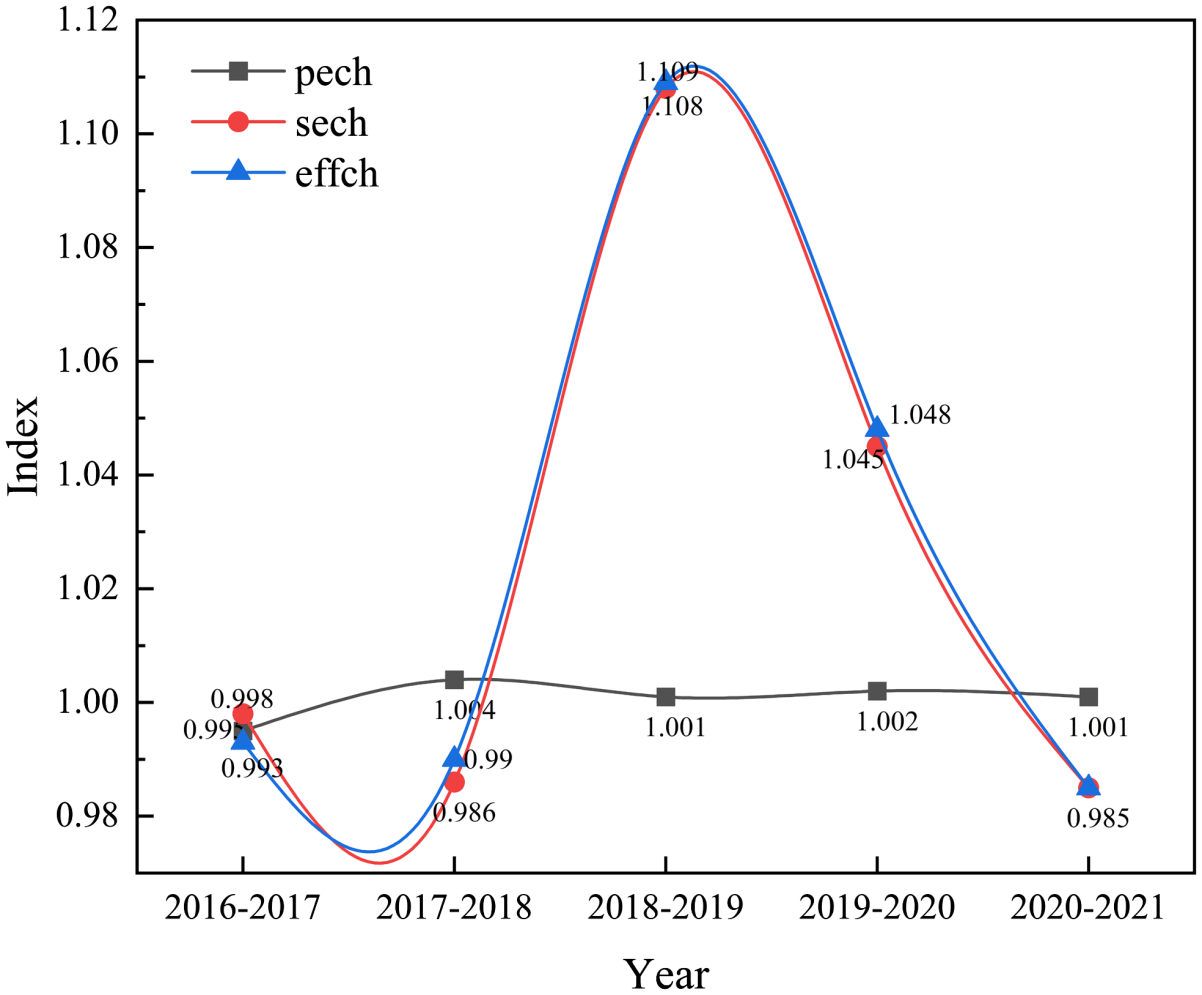
Changes in efficiency of maternal and child health care services from 2016 to 2021.

Efficiency changes can be decomposed into SECH and PECH. As shown in Figure 4, from 2016 to 2021, the efficiency change index exhibited a fluctuating downward trend. However, the efficiency change index was greater than 1 in 2018-2019 and 2019-2020, indicating an increasing trend in efficiency change during this period. On the other hand, the efficiency change indices from 2016 to 2018 and from 2020 to 2021 were both less than 1, indicating a downward trend in efficiency changes during these periods. The decomposition of efficiency reveals a consistent trend between efficiency change and SECH, suggesting that the decline in pure technical efficiency from 2016 to 2021 has somewhat slowed down the rate of efficiency change. The main driver of efficiency change is attributed to the SECH during the period.

### Malmquist index analysis of maternal and child health services in different regions

**Figure 5.**
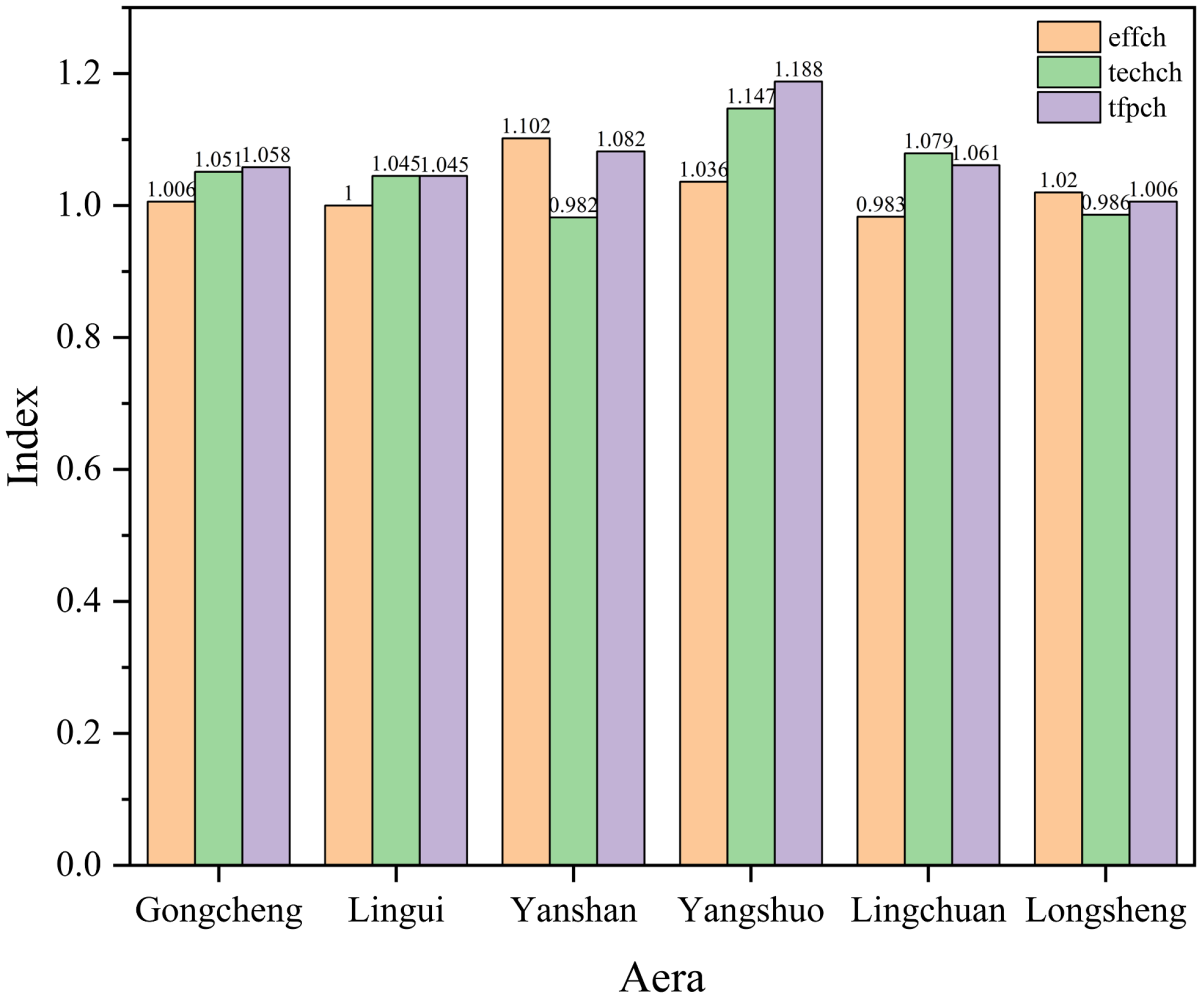
Changes in total factor productivity of maternal and child health services by regions from 2016 to 2021.

As shown in Figure 5, the total factor productivity index in all regions is greater than 1. This indicates that the allocation efficiency of MCH resources in each region is generally on an upward trend. An examination of the components of efficiency reveals that Gongcheng County, Lingui District, and Yangshuo County had both efficiency changes and technological changes greater than 1, suggesting that the increase in efficiency in these areas resulted from the combination of both factors. The efficiency change in Yanshan District and Longsheng County is greater than 1, indicating that the increase in the total factor productivity index is primarily due to improvements in the efficiency change index. Lingchuan County had a technological change index greater than 1, indicating that the increase in efficiency was primarily due to advancements in the technological change index.

**Figure 6.**
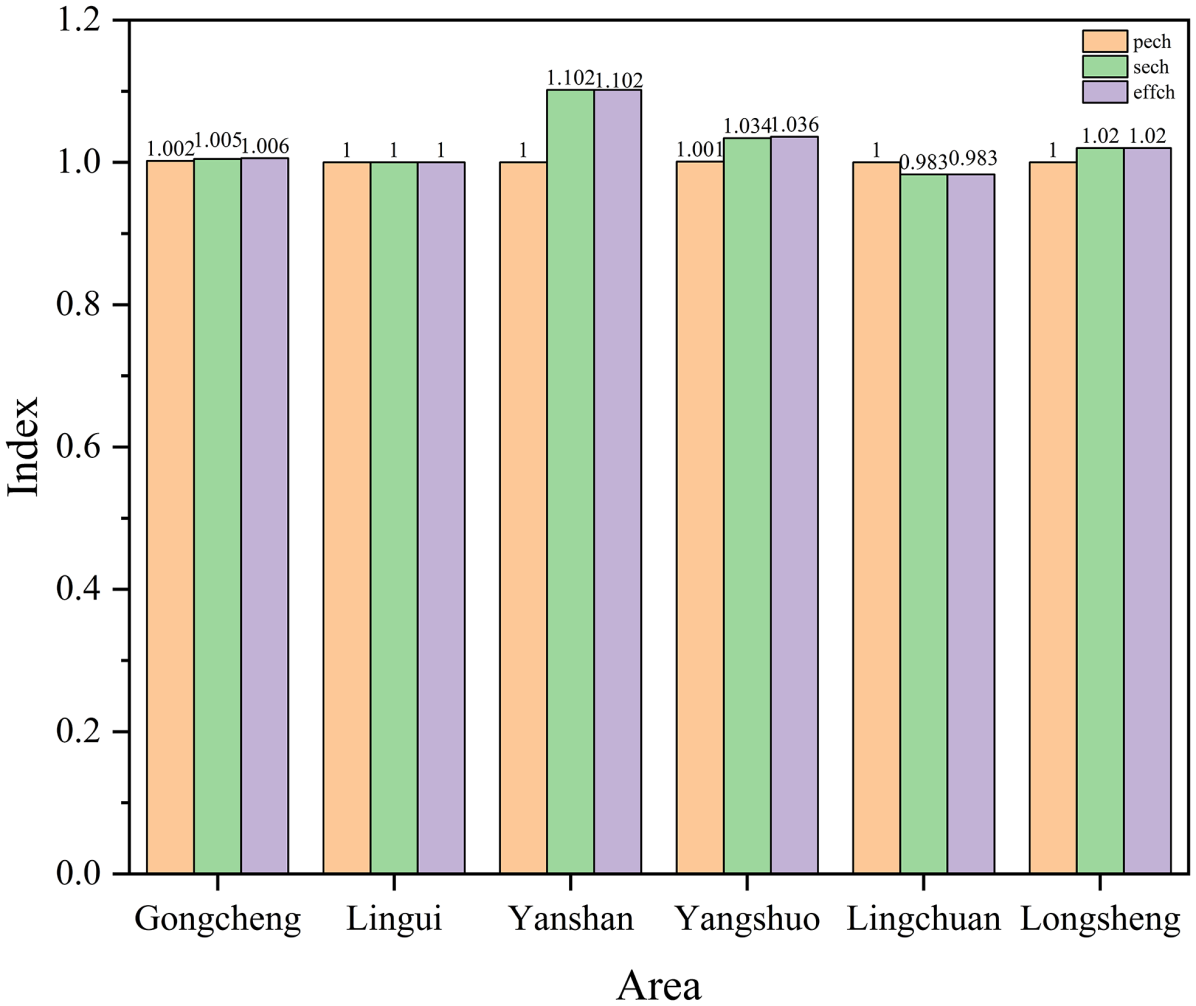
Changes in efficiency of maternal and child health care services by regions from 2016 to 2021.

As shown in Figure 6, an analysis of the efficiency changes from 2016 to 2021 reveals that the efficiency change indices for Gongcheng County, Yanshan District, and Longsheng County are all greater than 1, indicating an overall upward trend in efficiency changes. Lingchuan County had an efficiency change index less than 1, indicating an overall downward trend in efficiency changes. An examination of the components of efficiency reveals that Gongcheng County and Yangshuo County had both pure technical efficiency and scale efficiency greater than 1, indicating that the improvement in efficiency changes was driven by simultaneous increases in both pure technical efficiency and scale efficiency. Lingchuan County had a pure technical efficiency change equal to 1 and a SECH index less than 1, suggesting that its decrease in efficiency was primarily due to a decline in scale efficiency.

## Discussion

In 2009, according to the policies of the new healthcare reform, which aimed to “ensure that urban and rural residents have equal access to the most basic and effective public health services and to narrow the urban-rural gap,” China increased its investment in rural health care projects. Local governments strengthened the capacity building of rural health care institutions through urban-rural coordination (Wang *et al.*, 2020; Zhou *et al.*, 2024). This study shows that MCH services in township health centers have overall improved. However, the incidence of birth defects and low birth weight has been on the rise. These findings are consistent with studies conducted in Heilongjiang Province, China (Tian *et al.*, 2022), and Ethiopia, Africa (Kassaw *et al.*, 2021). It is reported that approximately five million infants with birth defects are born globally each year, with over 85% of these cases occurring in developing countries (Zhong and Jiang, 2019), and nearly 15% of babies worldwide are low birth weight and more than half of them are born in Asia (Kouser *et al.*, 2020). Environmental and biological factors, along with social progress leading to increased detection rates of congenital abnormalities, continuous improvements in prenatal examinations and screening methods, enhanced diagnostic levels and quality, improved tertiary surveillance networks, and an increase in hospital deliveries (Findley *et al.*, 2023; Fomda *et al.*, 2023; Huang *et al.*, 2021) all contribute to the causes of this rise. Additionally, recent studies have suggested a potential association between the occurrence of low birth weight and maternal exposure to air pollution during the preconception (Bekkar *et al.*, 2020) and early pregnancy periods, as well as the supply of nutrients during pregnancy (Harper *et al.*, 2023). Therefore, through the implementation of a special management model for high-risk infants and young children, a planned management approach is adopted to identify and monitor high-risk cases, ensuring early detection, evaluation, diagnosis, and intervention for developmental abnormalities and deformities.

According to the entropy weight coefficient method, we found that there are still disparities in MCH services across different regions. Longsheng County and Gongcheng County perform poorly in MCH services, showing significant room for improvement compared to other areas. First, Longsheng County and Gongcheng County are ethnic minority areas that face a scarcity of MCH service resources. For example, Lingui District, which performs well in MCH services, ranks second in economic level within the city. It is a directly governed district with relatively abundant MCH resources, and it is close to several municipal public hospitals and MCH centers. Other studies have found that regions with strong economic development and abundant healthcare resources often exhibit better MCH services (Paul *et al.*, 2022; Rios Quituizaca *et al.*, 2021). Therefore, the differences in the capacity of MCH services among regions may be attributed to a combination of varying economic levels (Janevic *et al.*, 2020)and the strength of MCH capabilities in each area (Ramadan *et al.*, 2023). Second, township health centers in minority counties are often located in remote mountainous areas with inconvenient transportation, which may lead to difficulty in covering all populations in need (Kaiser and Barstow, 2022). Third, there is a loss of talent in MCH. Numerous studies have demonstrated that satisfaction with compensation is a significant factor that affects the professional loyalty of primary healthcare workers (Soesanto *et al.*, 2022; Zhang *et al.*, 2020). Compared to clinical doctors, MCH physicians earn significantly lower salaries (Brekke *et al.*, 2020), leading them to pursue higher wages in clinical departments, which results in a decline in the quality of MCH services. Therefore, it is necessary to improve transportation and raise the salaries of MCH workers so that they can better serve women and children in rural areas.

In the efficiency analysis of MCH services, we found that all years, except for 2016 and 2018, were in a DEA inefficient state. Specifically, over 60% of the areas demonstrated low efficiency in MCH, with a decline in scale efficiency, indicating that these inefficient regions are using more healthcare resources than currently needed for health services. According to the input slack data, almost all inefficient regions had input redundancies, particularly in the number of medical equipment (≥CNY 10,000) (Chinese Yuan). Several factors could explain this outcome. One possibility is that the demand for MCH services was overestimated, resulting in redundant MCH resources in these inefficient regions. While this chance may be minimal, it should not be overlooked in decision-making. From the 1970s to the early 2000s, China’s health institutions gained considerable autonomy with limited government subsidies during the market-oriented reform period (Huang *et al.*, 2020). Influenced by market competition, these institutions significantly increased investments such as facility expansion, bed installation, and medical equipment purchases (Jiang and Pan, 2020). A previous survey indicated that less than 50% of large-scale medical devices were in use in China (Huang *et al.*, 2020). This underutilization of invested resources can lead to significant waste, ultimately reducing overall MCH efficiency. This indicates that health administrators should be aware of potential resource redundancy when formulating regional health plans.

Another possibility is that many families prefer municipal MCH hospitals to local primary health service centers in search of higher-quality MCH services, as municipal tertiary hospitals typically have more skilled healthcare personnel and better technology (Zhou *et al.*, 2024). This phenomenon suggests a certain level of inequality in the allocation of MCH resources and healthcare utilization, with urban areas having more experienced specialists and advanced equipment than rural areas. Sharing training programs at municipal MCH hospitals could be a potential way to improve the quality of healthcare services in underdeveloped rural health centers (Helmyati *et al.*, 2022). It may be useful to improve the quality of medical care in economically disadvantaged rural areas in a sustainable manner that grassroots health workers return to work after a period of receiving the technical training from municipal tertiary hospitals. For regions with inefficiencies, transferring their excessive input resources to more available health services for mothers and children is another reasonable method to improve the efficiency of MCH resources. The disparities in MCH performance across various regions may contribute to the observation that, at equivalent input levels, the volume of services generated by MCH in less efficient areas is noticeably lower than that in their more efficient counterparts. In this regard, attention should be directed toward enhancing the capacity for improved service delivery. The inefficient regions are primarily tasked with improving staff performance and the quality of their management practices in order to effectively utilize inputs and provide available health care services for the mothers and children in economically disadvantaged rural areas (Alotaibe *et al.*, 2022).

A study and analysis of panel data from different regions between 2016 and 2021 using the Malmquist index revealed varying degrees of improvement in TFPCH. Overall, there was a dynamic growth trend in TFPCH changes, which is consistent with the findings of Junxu Zhou’s analyzing the efficiency of Chinese primary healthcare institution (Zhou *et al.*, 2023). Zhang’s analysis based on the Malmquist index showed an average annual growth rate of 2.5% in the total factor productivity of county-level MCH institutions in China from 2010 to 2014 (zhang *et al.*, 2017). These studies all support the findings of the present research. The reasons for this trend could be attributed to the impact of policies such as the “New Medical Reform,” the “Outline for the Development of Women and Children,” and the “Decision on Optimizing Birth Policies to Promote Long-Term Balanced Population Development,” which have led to an overall increase in healthcare resource inputs. The TFPCH can be broken down into the TECHCH and the EFFCH, with the EFFCH further divided into PECH and SECH (Zhang *et al.*, 2023). However, our study revealed that TFPCH growth varied across regions. This also validates the evaluation of MCH by entropy weight coefficient to a certain extent. The growth in TFPCH is primarily driven by an increase in the EFFCH, while the rise in EFFCH is mainly due to the improvement in PECH. Guangxi has set up a four-level MCH service system covering provinces, cities, counties, and townships, and implemented a training program for MCH personnel, which included training targeted at admitting medical students to work in rural areas after graduation and strengthening the training of registered nurses (Zhou *et al.*, 2024). In contrast, changes in SECH may be the primary factor contributing to the decline in EFFCH, as seen in Longsheng County. Therefore, to improve the efficiency of MCH and support the sustainable growth of MCH services, Guangxi needs to further improve its internal structure and management levels to enhance scale efficiency. It is also essential to strengthen the service capabilities of primary health institutions (Ahmed *et al.*, 2019), promote collaboration among medical facilities at various levels (Lal *et al.*, 2021).

There are two limitations in this study. Firstly, Due to the ongoing *COVID-19* pandemic during the 2022 survey, there may have been complexities and challenges in grassroots MCH care services. The data related to grassroots MCH services in this survey were based on the Outline for the Development of Women and Children, the Service Evaluation Guide for Township Health Centers, and Basic Public Health Services. Discrepancies may exist compared to the actual work statistics and assessment indicator systems. Secondly, due to limitations in time and resources, the survey’s comprehensiveness might be limited. Future research endeavors aim to broaden the scope of data collection, conduct further investigations, refine data structures, elaborate on indicators, and further strengthen empirical research on grassroots medical and health systems.

## Conclusion

The new healthcare reform policies have catalyzed the development of primary healthcare institutions in our country, offering decision-making support for their rational and effective progress. This is particularly evident in the advancement of township health centers. The findings of this study hold significant implications for the further enhancement of primary MCH in rural China, as well as for the strategic planning of healthcare systems, especially in the less developed regions. This study conducted an empirical investigation into the current status, efficiency, and trends of MCH services in township hospitals in rural ethnic minority areas. The results indicate that, first, there has been an overall improvement in the level of MCH services. Furthermore, there has been a modest overall increase in the use of MCH, despite concerns about the increasing inaccessibility of MCH services in rural China. However, attention should be paid to the rising trend in the incidence of birth defects and low birth weight. Second, there are regional disparities in efficiency evaluation, which are related to the capacity of MCH services and the economic levels of each area. Experiences from other countries support this view. Third, the improvement in productivity has relied solely on technological advancements rather than enhancements in internal management and institutional innovation. Although the input of various resources has certainly contributed to improvements in MCH, the enhancement of SE has not kept pace. In the future, internal management should be strengthened by setting service goals and updating management concepts. Existing resources should be fully and rationally utilized, and the relationship between scaling up and improving quality should be addressed through the flow of resources among institutions.

## Data Availability

The datasets generated and/or analyzed during the current study are not publicly available due protect confidentiality of data from primary healthcare institutions but are available from the corresponding author on reasonable request. The formulas for data calculations in the appendix were also obtained from the corresponding author upon reasonable request.

## Appendix

### Entropy weight coefficient method

**Step 1:** Construct the corresponding output data matrix *X*. This converts original data into relative values between 0 and 1, where values closer to 1 indicate better performance. Indicators are classified as positive (higher values mean better performance) or negative (higher values mean worse performance):

**Step 2:** Calculate the weight P_*ij*_ of the *j*-th indicator for the *i*-th evaluation criterion:

**Step 3:** Calculate the entropy e_*ij*_ of the *j*-th indicator:

**Step 4:** Calculate the redundancy degree d_*j*_ of the entropy:

**Step 5:** Calculate the objective weight w_*j*_ for each indicator:

**Step 6:** Calculate the comprehensive score s_*j*_ for each evaluation unit:

**BCC**-**DEA Model**

The study employs the input-oriented BCC-DEA model, which can be expressed as:

In this expression, *θ* is the efficiency of DMU_*j*_, λ_*j*_ is the unit’s weight, *X*_*j*_ and *Y*_*j*_ are the input and output quantities of the _j_-th unit, *n* is the number of units, and S^+^ and S- are the input and output slack variables. The effectiveness of DMU_*j*_ is determined by the model’s optimal solution values.

(1)If θ=1 and both S ^+^ and S ^-^ are 0, DMU_*j*_ is DEA efficient; (2) If *θ*=1 but S ^+^ or S ^-^ is not 0, DMU_*j*_ is weakly DEA efficient; (3) If *θ*<1 and S ^+^ or S - is not 0, DMU_*j*_ is non-DEA efficient.

### Malmquist index Model

The MPI, also known as Total Factor Productivity Changes (TFPCH), is derived from the distance function and can be represented by the following mathematical equations:

To thoroughly understand the technical level during both periods, we took into account the geometric mean:

The productivity function includes input-oriented efficiency change (EFFCH) and technical change (TECHCH). Efficiency change can also be divided into scale efficiency change (SECH) and pure efficiency change (PECH).
